# Hemolysin III drives neuroinvasion and meningitis while impacting desiccation resilience in *Cronobacter sakazakii*

**DOI:** 10.1128/aem.00339-25

**Published:** 2025-03-05

**Authors:** Jiawei Liu, Qianquan Wan, Daizong Gaoxin, Zhanhui Song, Ke Mao, Fengzhu Wu, Xiangyu Liu, Linsen Mu

**Affiliations:** 1 Department of No.9 Neurosurgery, Guangdong Sanjiu Brain Hospital 639524 , Guangzhou, China; The Pennsylvania State University, University Park, Pennsylvania, USA

**Keywords:** *Cronobacter sakazakii*, Hemolysin III (Hly III), neuroinvasion, blood-brain barrier (BBB), meningitis, environmental resilience

## Abstract

*Cronobacter sakazakii* is an emerging pathogen associated with severe neonatal diseases, including meningitis. Its ability to cross the blood-brain barrier (BBB) is critical for meningitis development, yet the underlying mechanisms remain unclear. This study investigates the role of Hemolysin III (Hly III), encoded by the *ESA_00432*, in neuroinvasion and environmental resilience. A markerless deletion of *ESA_00432* (Δ*ESA_00432*) revealed that the mutant exhibited unaltered biofilm formation, increased hydrophilicity, and enhanced desiccation resistance compared to the wild type, suggesting that Hly III imposes a fitness cost on *C. sakazakii* under non-invasive conditions. In a rat infection model, the Δ*ESA_00432* strain demonstrated significantly reduced brain colonization without affecting bacterial loads in blood, liver, or spleen, underscoring the specific importance of Hly III in neuroinvasion. Cellular assays further revealed that, although the mutant maintained similar levels of adherence and invasion in Caco-2 cells and comparable adhesion to human brain microvascular endothelial cells (HBMECs) as the wild type, its ability to invade HBMECs was markedly diminished. These results suggest that Hly III is crucial for efficient neuroinvasion and BBB translocation while imposing a trade-off on environmental resilience, providing insights into the balance between virulence, and environmental adaptability in *C. sakazakii*.

**IMPORTANCE:**

The ability of *Cronobacter sakazakii* to cause severe neonatal infections, particularly meningitis, presents a significant public health concern, yet the molecular mechanisms that enable its neuroinvasion remain poorly understood. In this study, we identify Hemolysin III (Hly III), encoded by the ESA_00432 gene, as a key factor in the bacterium’s ability to cross the blood-brain barrier (BBB) and initiate meningitis. Our findings demonstrate that Hly III is essential for efficient invasion of human brain microvascular endothelial cells (HBMECs) and subsequent brain colonization in a rat model, underscoring its critical role in neurotropism. Furthermore, we show that the absence of Hly III results in enhanced environmental resilience, as indicated by increased desiccation resistance and hydrophilicity. This metabolic trade-off between virulence and environmental adaptability reveals a novel aspect of *C. sakazakii*’s pathogenesis and survival strategies. These insights open new avenues for developing targeted interventions to prevent neonatal meningitis and enhance food safety measures against this opportunistic pathogen.

## INTRODUCTION

*Cronobacter sakazakii* (formerly *Enterobacter sakazakii*) is an opportunistic foodborne pathogen that poses a significant threat to neonates, particularly those with low birth weight or compromised immune systems (1). It is associated with severe infections, including neonatal meningitis, sepsis, and necrotizing enterocolitis, with reported mortality rates reaching up to 27% (2). Survivors often experience long-term neurological complications such as hydrocephalus, quadriplegia, and impaired cognitive development, underscoring the devastating consequences of *C. sakazakii* infections (3).

A major concern in food safety, *C. sakazakii* is primarily transmitted through contaminated powdered infant formula (PIF), where it exhibits exceptional resistance to desiccation, enabling long-term survival in dry environments (4). Studies have shown that *C. sakazakii* can persist for years in PIF and production facilities, posing an ongoing risk of contamination (5–7). Beyond PIF, cases of infection linked to contaminated breast milk and hospital equipment suggest that the pathogen has multiple transmission routes (8–10), further complicating infection control efforts. Once ingested, *C. sakazakii* colonizes the intestinal tract, breaches the intestinal epithelium, and disseminates systemically, ultimately crossing the blood–brain barrier (BBB) to cause meningitis (10).

A hallmark of *C. sakazakii* pathogenesis is its ability to invade human brain microvascular endothelial cells (HBMECs) and traverse the BBB via transcytosis, a critical step in the development of neonatal meningitis (11, 12). Several bacterial factors have been implicated in this process, including outer membrane proteins OmpA and OmpX, which mediate adhesion and invasion of HBMECs (13). Additionally, the RNA chaperone Hfq and the *Cronobacter* plasminogen activator facilitate immune evasion and systemic dissemination (14, 15).

While these virulence factors are well characterized, the role of hemolysins in *C. sakazakii* pathogenesis remains poorly understood. Hemolysins are pore-forming toxins that disrupt host cell membranes, facilitating bacterial invasion, immune evasion, and tissue damage in a range of bacterial pathogens (16–18). In *Escherichia coli*, α-hemolysin aggravates BBB disruption by interfering with TGFβ1-activated Hedgehog signaling, thereby promoting central nervous system (CNS) infections (19). Similarly, *Listeria monocytogenes* utilizes listeriolysin O to escape vacuolar compartments, enabling intracellular survival and BBB invasion (20). Given the role of hemolysins in other neuroinvasive pathogens, *C. sakazakii* hemolysins, particularly Hemolysin III (Hly III), may play a previously unrecognized role in BBB translocation and meningitis development.

In this study, we investigate the contribution of Hly III to *C. sakazakii* pathogenesis, focusing on its role in BBB invasion and disease progression. Using a rat infection model and *in vitro* HBMEC assays, we assess the impact of *hly* deletion on bacterial adherence and invasion efficiency. Furthermore, we explore the metabolic trade-offs associated with Hly III expression, providing new insights into its role in bacterial fitness and virulence regulation. These findings will contribute to a deeper understanding of *C. sakazakii* pathogenesis and may inform future strategies for infection control and prevention.

## RESULTS

### Construction and growth characteristics of the *ESA_00432* mutant in *C. sakazakii*

**Fig 1:**
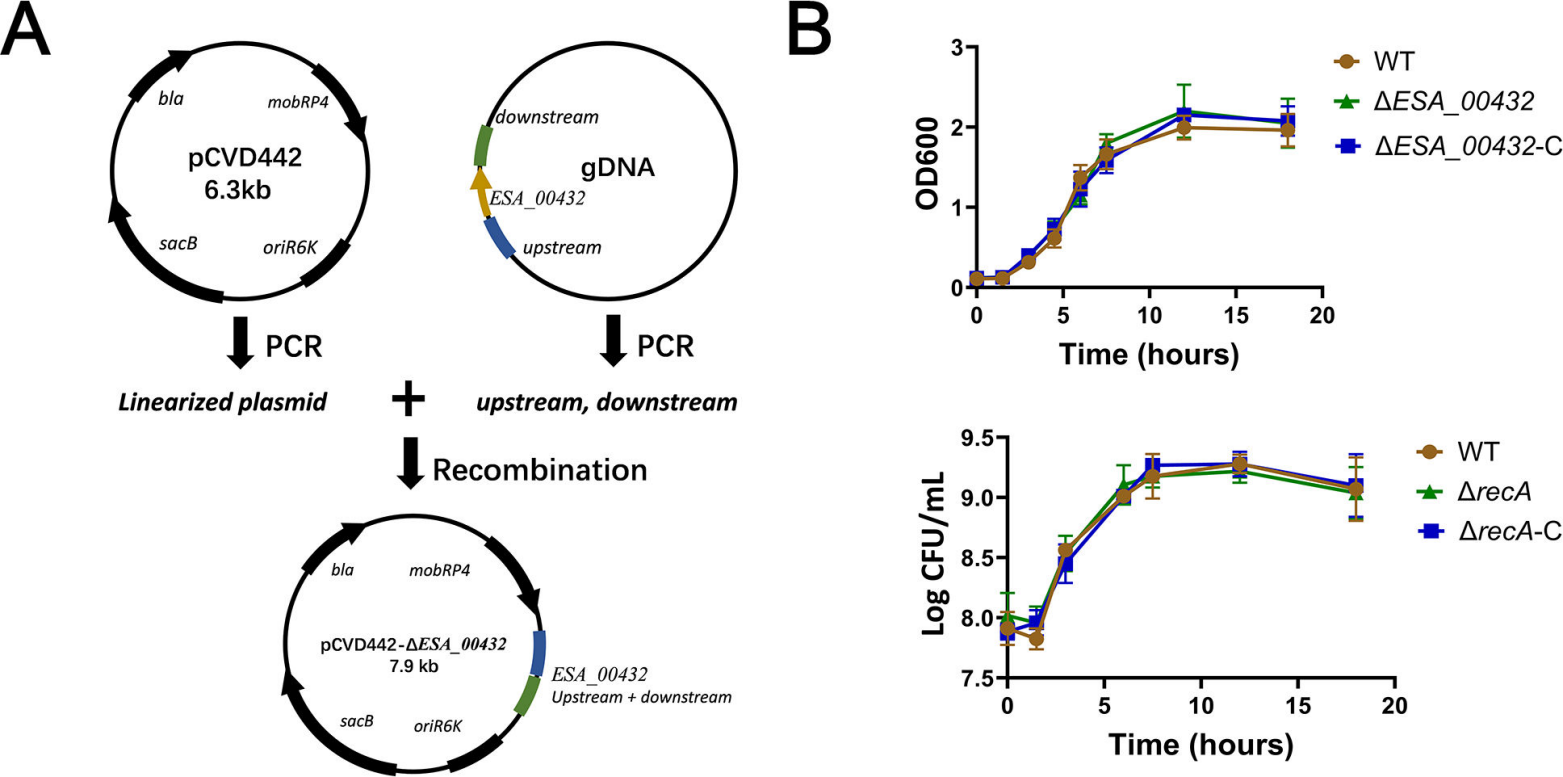
Construction and growth characteristics of the *ESA_00432* mutant in *Cronobacter sakazakii*. (**A**) Schematic representation of the construction of the *ESA_00432* gene knockout mutant using the pCVD442 suicide plasmid system. Approximately 800 bp upstream and downstream flanking regions of ESA_00432 were amplified by PCR and cloned into the linearized pCVD442 plasmid. The recombinant plasmid was introduced into *C. sakazakii* via biparental conjugation and in-frame deletion mutants were selected using sucrose counter-selection. (**B**) Growth curves of wild type (WT), Δ*ESA_00432* mutant, and the complemented strain (Δ*ESA_00,432-*C) were monitored in LB medium at 37°C. Optical density (OD_600_) and colony-forming units (CFU/mL) were measured over 20 hours. Statistical differences among strains were analyzed using one-way analysis of variance (ANOVA) followed by Tukey’s post hoc test. Error bars represent the standard deviation of three biological replicates, and significance was set at *P* < 0.05.

To investigate the role of the *ESA_00432* gene, which encodes Hemolysin III, in the environmental adaptability and pathogenicity of *C. sakazakii* ATCC BAA-894, an *ESA_00432* knockout mutant (Δ*ESA_00432*) was constructed using the pCVD442 suicide plasmid technique (Fig. 1A). Successful deletion of the *ESA_00432* gene was confirmed through PCR and sequencing analysis. Growth curve analysis in the Luria-Bertani (LB) medium at 37°C showed no significant differences in growth rate between the Δ*ESA_00432* mutant and the wild-type strain. Additionally, the introduction of the *ESA_00432* complementation plasmid did not alter the growth characteristics, confirming that the deletion of *ESA_00432* does not impact the growth of *C. sakazakii* under standard laboratory conditions (Fig. 1B). These findings suggest that Hly III is not required for growth in rich media.

### *ESA_00432* mutation improves survival without affecting systemic bacterial load

**Fig 2:**
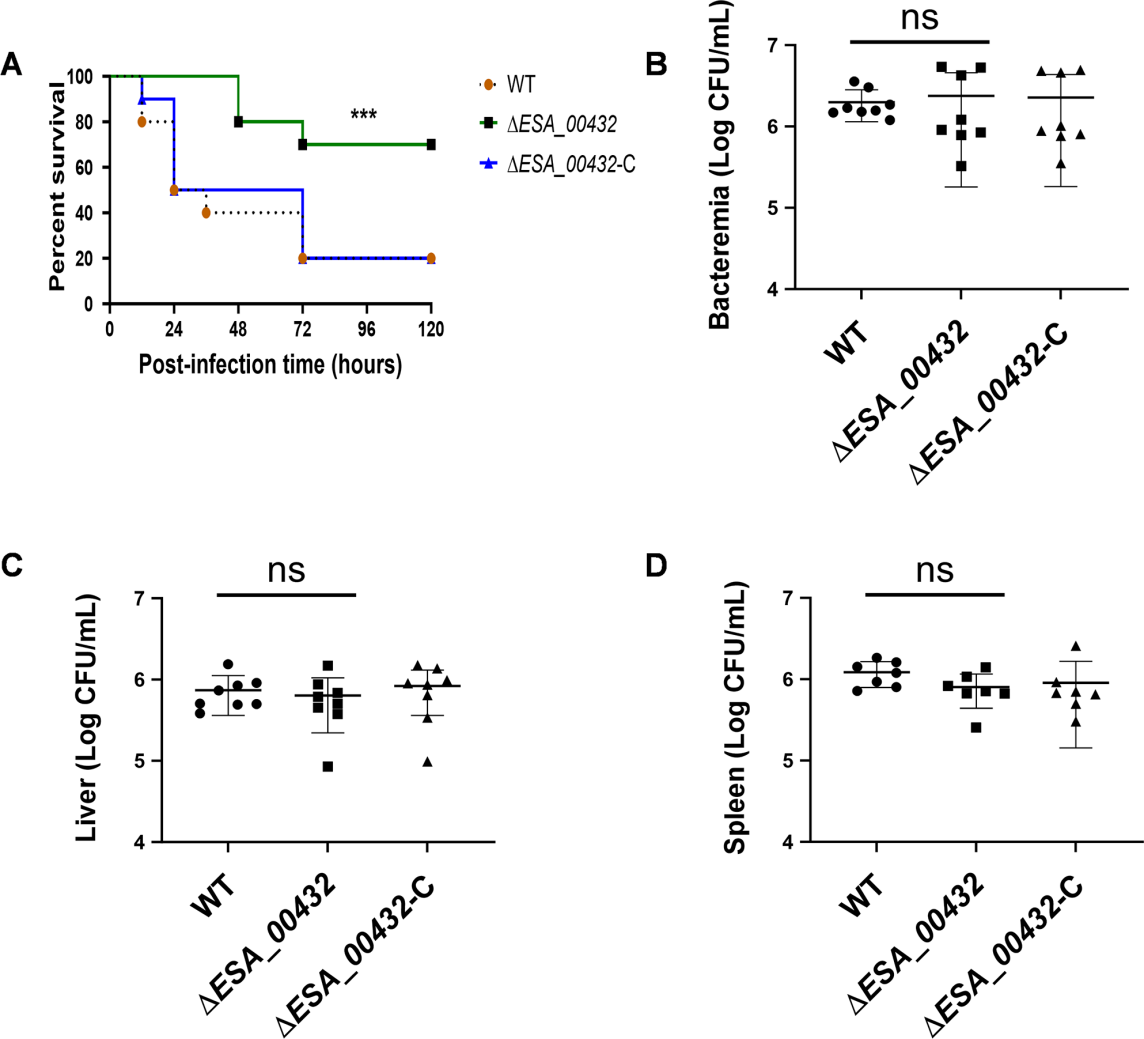
Survival and systemic bacterial load following infection with *ESA_00432* mutant strains. (**A**) Survival curves of Sprague-Dawley rat neonates intragastrically administered with 1 × 10⁸ CFU of WT, Δ*ESA_00432* mutant, or Δ*ESA_00432* complemented strain in 20 µL of sterile phosphate-buffered saline (PBS). Survival differences were analyzed using the log-rank test. (B–D) Bacterial loads in blood (**B**), liver (**C**), and spleen (**D**) at 24 hours post-infection. CFU counts were determined to assess systemic dissemination. Statistical comparisons among groups were performed using one-way ANOVA. Data are presented as mean ± standard deviation (*n* ≥ 6 rats per group), and significance was set at *P* < 0.05.

Rats were infected with wild type (WT), Δ*ESA_00432* mutant, and Δ*ESA_00432* complemented strains, and their survival was monitored over 7 days. A significant difference in survival curves was observed between rats infected with the Δ*ESA_00432* mutant and those infected with the WT strain. Rats infected with the Δ*ESA_00432* mutant exhibited a markedly higher survival rate, particularly within the first 5 days post-infection, whereas the survival rate of rats infected with the Δ*ESA_00432* complemented strain was restored to levels similar to the WT strain (Fig. 2A). The bacterial loads in the blood, liver, and spleen of infected rats were assessed 24 hours post-infection by homogenizing the tissues, culturing the samples, and determining colony counts. The results revealed no significant differences in bacterial loads between the WT strain, Δ*ESA_00432* mutant, and Δ*ESA_00432* complemented strain in blood, liver, or spleen (Fig. 2B through D). These findings indicate that while the absence of Hly III does not affect systemic bacterial dissemination, it significantly reduces virulence, as reflected in the survival outcomes.

### Role of ESA_00432 in neuroinvasion and brain colonization in a rat infection model

**Fig 3:**
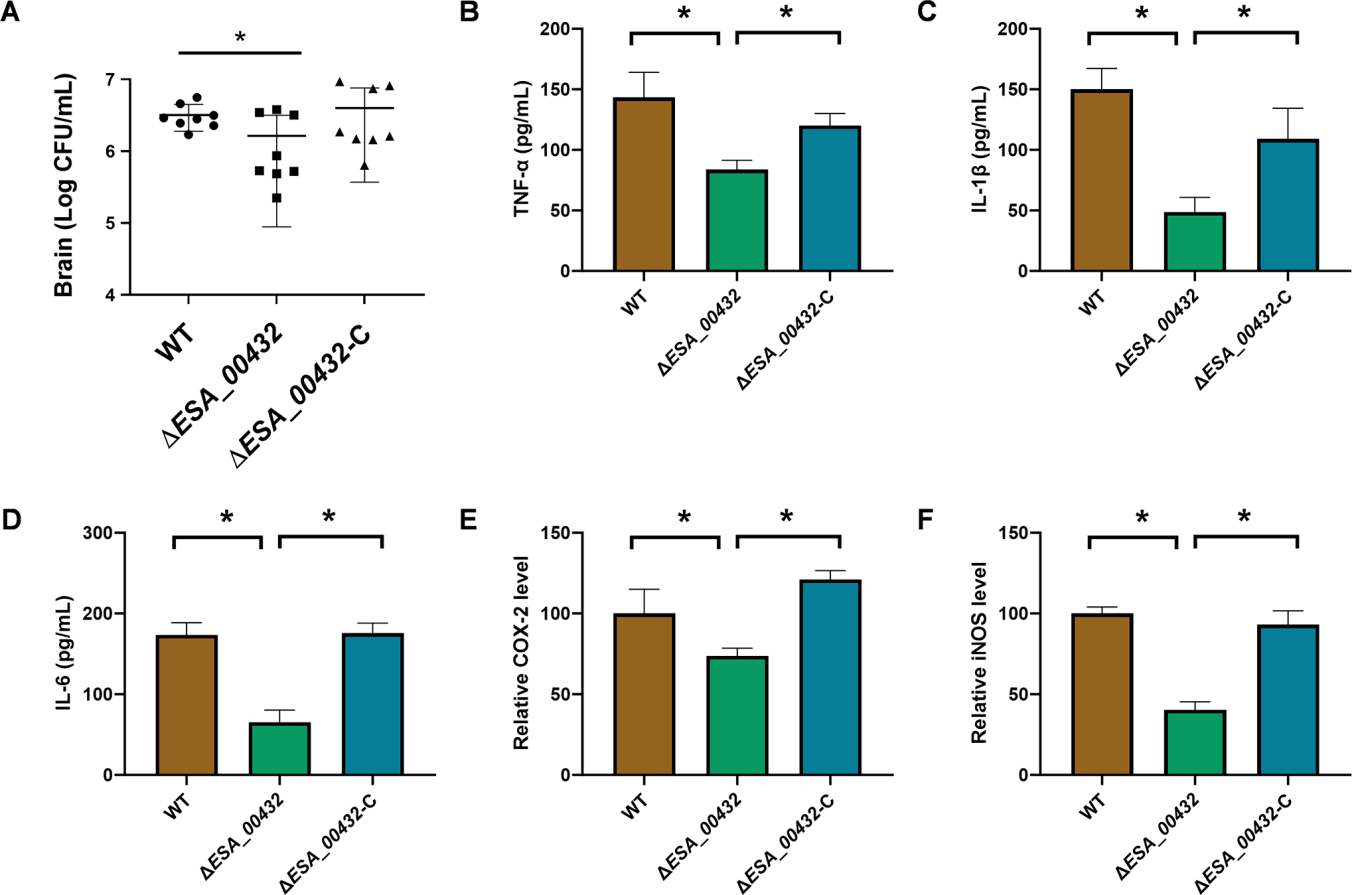
Neuroinvasion and brain inflammatory response associated with ESA_00432 mutation. (**A**) Bacterial loads in brain tissues of Sprague-Dawley rat neonates infected with WT, Δ*ESA_00432* mutant, or Δ*ESA_00432* complemented strain. (B–D) Levels of pro-inflammatory cytokines TNF-α (**B**), IL-1β (**C**), and IL-6 (**D**) in brain tissue homogenates, as measured by enzyme-linked immunosorbent assay. (**E and F**) Relative expression levels of COX-2 (**E**) and iNOS (**F**) in brain tissues. Statistical comparisons were performed using one-way ANOVA. Data are presented as mean ± standard deviation (*n* ≥ 6 per group), with significance set at *P* < 0.05.

The improved survival of rats infected with the Δ*ESA_00432* mutant, despite no changes in systemic bacterial load, prompted further investigation into its ability to invade and colonize brain tissue. Bacterial loads in brain tissues were significantly lower in rats infected with the Δ*ESA_00432* mutant compared to those infected with the wild-type strain (Fig. 3A), indicating impaired neuroinvasion. Additionally, the inflammatory response in the brain was assessed by measuring levels of pro-inflammatory cytokines (TNF-α, IL-1β, IL-6) and enzymes (COX-2, iNOS). Rats infected with the Δ*ESA_00432* mutant showed significantly reduced levels of these inflammatory markers compared to the WT strain (Fig. 3B through F). The Δ*ESA_00432* complemented strain restored both brain bacterial loads and inflammatory marker levels to those observed in the wild-type group, confirming the role of Hly III in neuroinvasion and modulation of the brain’s inflammatory response.

### Impact of HLY III on cellular adherence and invasion

**Fig 4:**
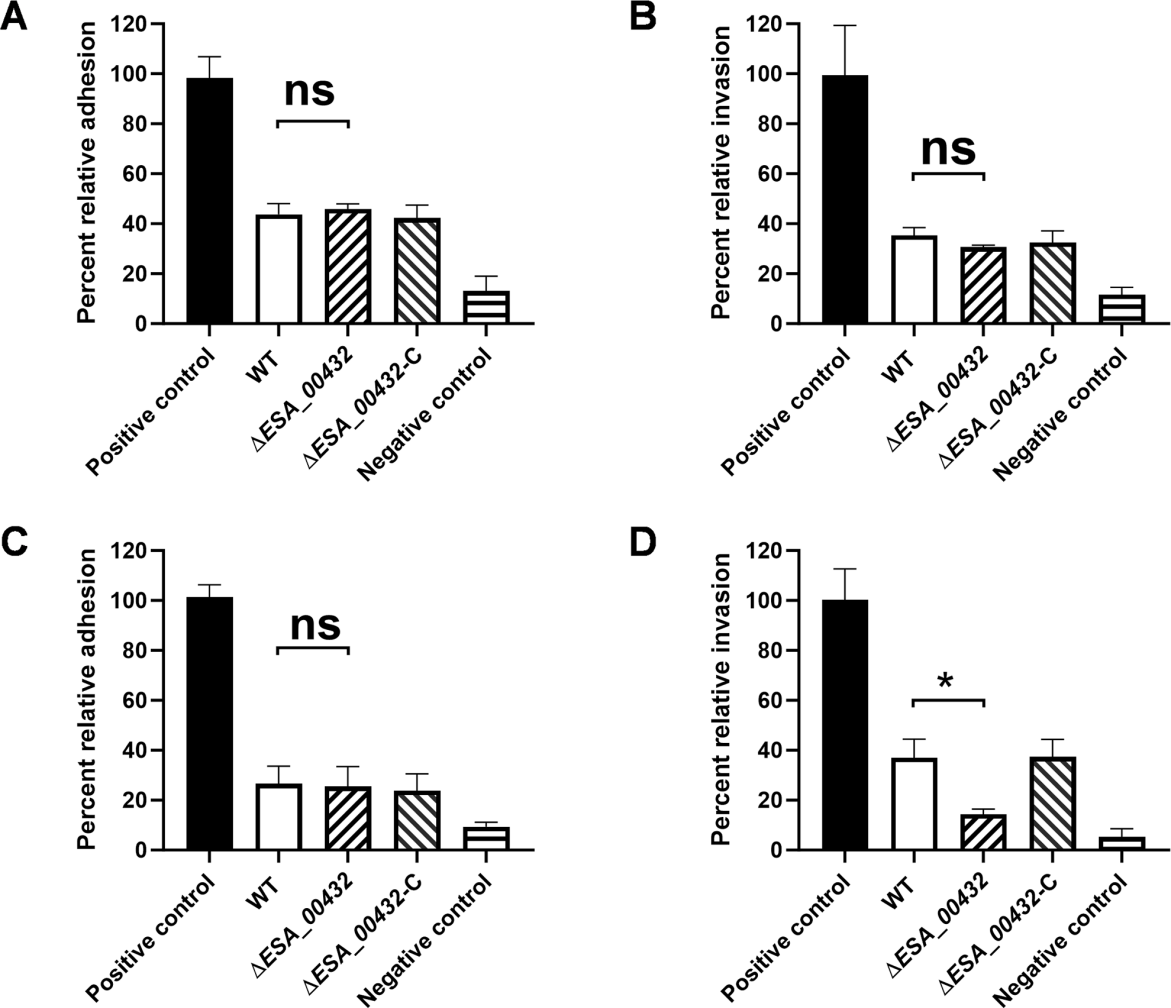
Impact of Hly III on cellular adherence and invasion. Cellular assays were conducted to assess the role of the *ESA_00432* gene in the adherence and invasion of *Cronobacter sakazakii* into human intestinal (Caco-2) and endothelial (HBMECs) cells. (**A and B**) The relative adhesion and invasion of *C. sakazakii* strains to Caco-2 cells, (**C and D**) The relative adhesion and invasion of *C. sakazakii* strains to HBMECs. The positive control used *Listeria monocytogenes*, and the negative control used *E. coli* DH5α. Data are presented as mean ± standard deviation (*n* = 3 per group), with significance set at *P* < 0.05.

Cellular assays were conducted to evaluate the impact of the *ESA_00432* gene on the adherence and invasion of *C. sakazakii* into human intestinal and endothelial cells. The Δ*ESA_00432* mutant demonstrated no significant difference in the adherence to or invasion of Caco-2 cells compared to the wild-type strain (Fig. 4A and B). Likewise, adherence to HBMECs was similar between the Δ*ESA_00432* mutant and the wild-type strain (Fig. 4C). However, a notable reduction in the ability of the Δ*ESA_00432* mutant to invade HBMECs was observed (Fig. 4D). The complementation of *ESA_00432* restored the invasion ability of HBMEC invasion. These findings indicate that while Hly III does not affect adherence to Caco-2 or HBMECs, it is essential for the efficient invasion of HBMECs.

### Biofilm formation and environmental stress resistance of the Δ*ESA_00432* mutant

**Fig 5:**
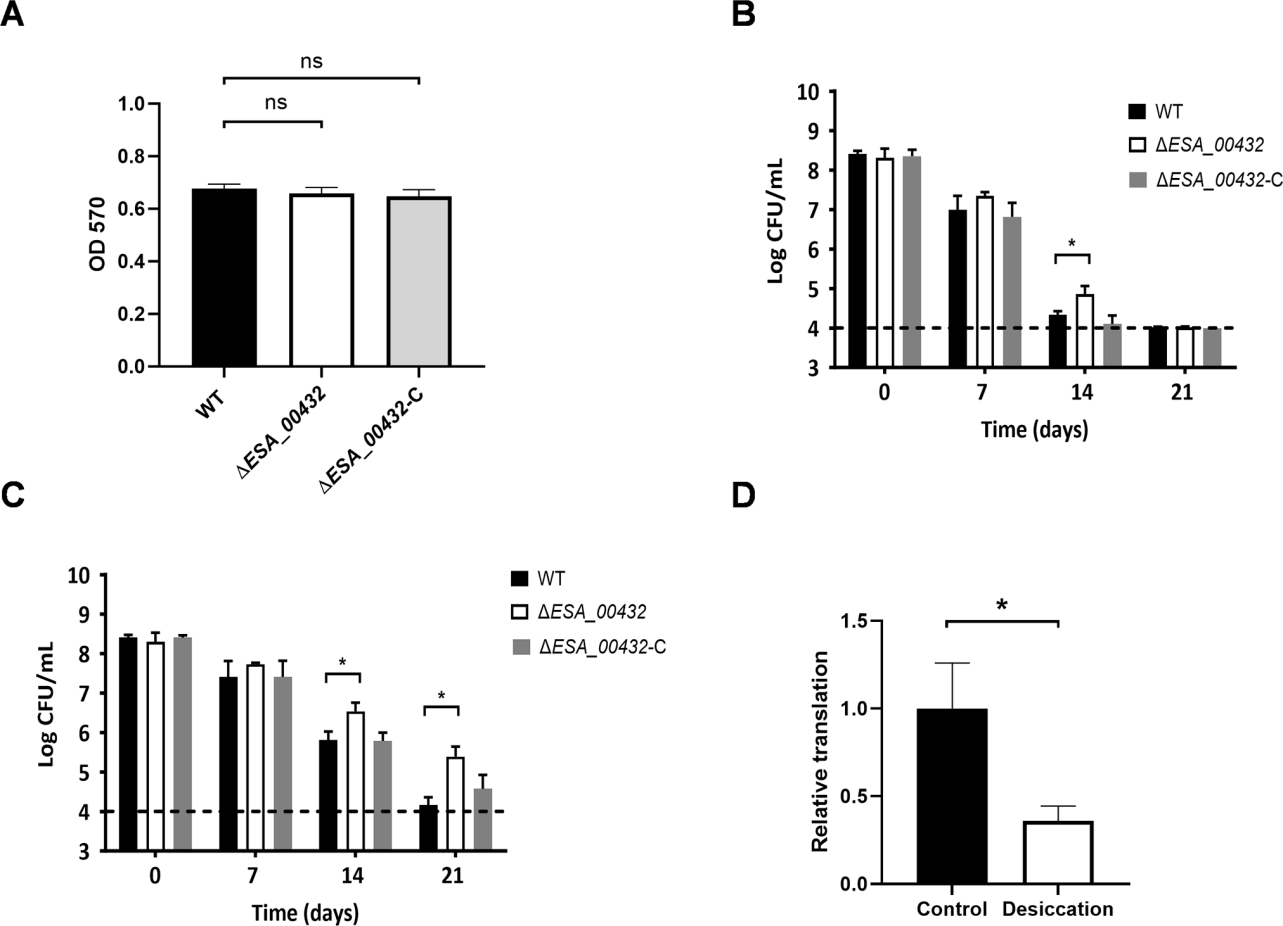
Biofilm formation and desiccation resistance of the Δ*ESA_00432*. (**A**) Biofilm formation assays under static conditions for the Δ*ESA_00432* mutant, WT, and Δ*ESA_00432* complemented strain. (**B and C**) Changes in viable cell counts (CFU/mL) during desiccation in PBS and milk powder, respectively. (**D**) Relative expression of the *ESA_00432* in the wild-type strain cultured in LB medium and samples subjected to desiccation for 3 days. Data are presented as mean ± standard deviation (*n* = 3 per group), with significance set at *P* < 0.05.

To further assess the role of Hly III in environmental resilience, biofilm formation and resistance to desiccation were evaluated. The Δ*ESA_00432* mutant was tested for its ability to form biofilms on polystyrene surfaces under static conditions. The results showed no significant difference in biofilm formation between the Δ*ESA_00432* mutant and the wild-type strain (Fig. 5A), suggesting that Hly III does not play a role in biofilm formation under these conditions. Additionally, the mutant’s resistance to desiccation was tested by drying bacterial cultures and subsequently rehydrating them to assess survival rates. Desiccation resistance assays revealed that the Δ*ESA_00432* mutant exhibited significantly enhanced survival rates compared to the wild-type strain when subjected to desiccation in both phosphate-buffered saline (PBS) and milk powder (Fig. 5B and C), indicating that the absence of Hly III may provide a survival advantage under environmental stress conditions. Furthermore, a significant reduction in the expression of the Hly III gene was observed in the wild-type strain under dry conditions (Fig. 5D), suggesting that *C. sakazakii* may enhance its desiccation tolerance by downregulating Hly III expression under these conditions.

### Hydrophilicity of the Δ*ESA_00432* mutant

**Fig 6:**
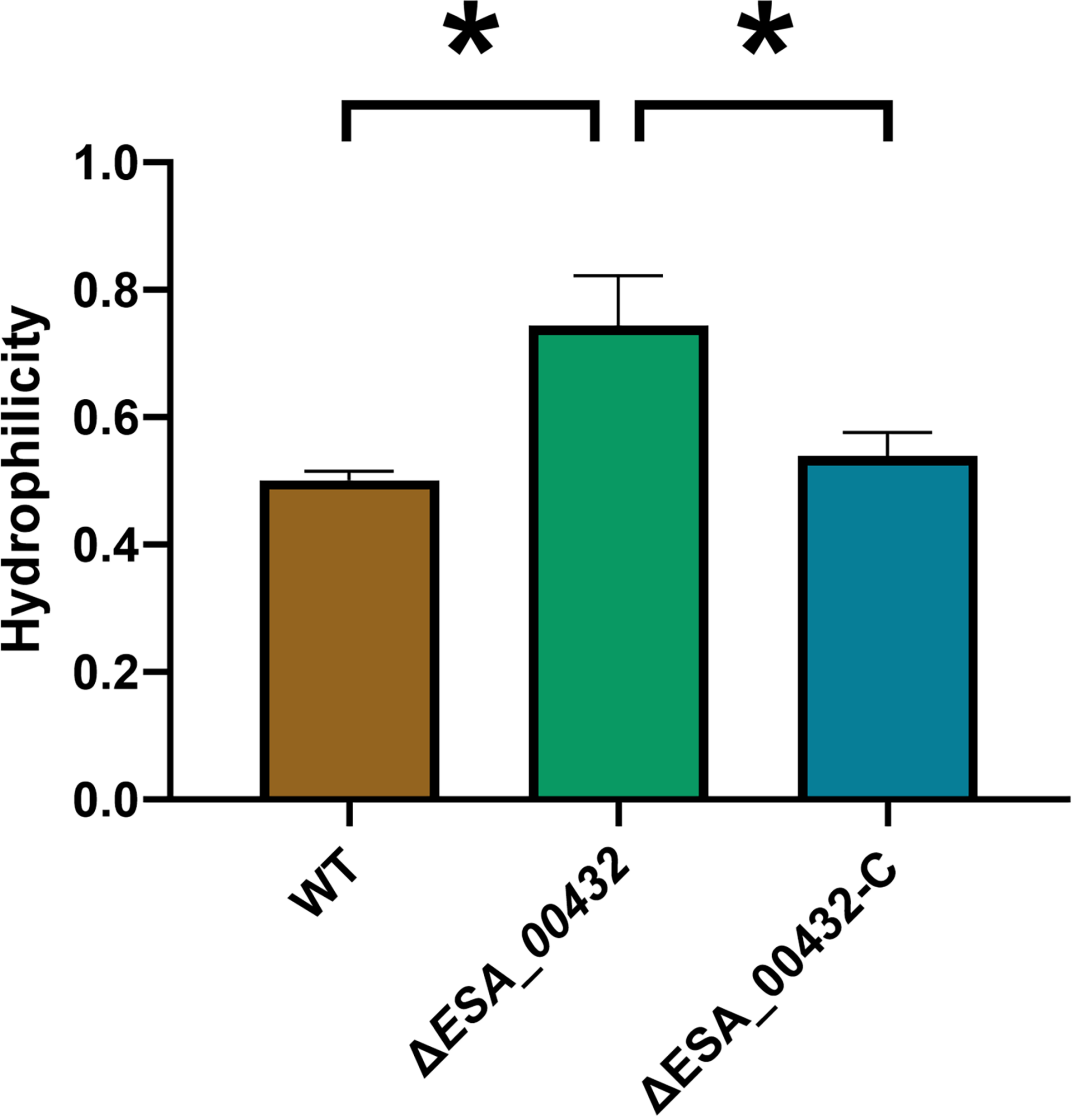
Hydrophilicity of the Δ*ESA_00432* mutant. The hydrophobicity was calculated based on the OD_600_ of the aqueous phase after phase separation, comparing the WT, ΔESA_00432 mutant, and complemented strains. Data are presented as mean ± standard deviation (*n* = 3 per group), with significance set at *P* < 0.05.

The impact of the *ESA_00432* mutation on surface properties, particularly hydrophilicity, was also investigated to understand its relationship with environmental stress tolerance. The hydrophilicity of the Δ*ESA_00432* mutant was measured using a xylene-water phase separation assay. The Δ*ESA_00432* mutant exhibited a significantly higher hydrophilicity compared to the wild-type strain, as indicated by an increased OD_600_ of the water phase (Fig. 6). This result suggests that the mutation enhances the hydrophilic surface properties of *C. sakazakii*, which could contribute to improved environmental survival, particularly under desiccation conditions. The observed increased hydrophilicity, in combination with enhanced desiccation resistance, underscores the trade-off between virulence factors and environmental resilience in the Δ*ESA_00432* mutant.

## DISCUSSION

The role of bacterial hemolysins in virulence has been well-documented across various pathogens. In *Escherichia coli*, hemolysin production contributes to brain colonization during meningitis (19). Similarly, in *Listeria monocytogenes*, the hemolysin LLO is critical for crossing the BBB and facilitating neuroinvasion (21). In this study, the lack of significant changes in bacterial loads in the blood, liver, and spleen of rats infected with the Δ*ESA_00432* mutant suggests that Hly III’s primary function is not in systemic dissemination, but rather in localized tissue invasion, particularly in the brain. This finding parallels studies in *Pseudomonas syringae*, where the absence of certain virulence factors impaired tissue-specific colonization without affecting overall systemic spread (22). Consistent with these observations, the reduced bacterial loads in brain tissue of the Δ*ESA_00432* mutant provide compelling evidence that Hly III is crucial for BBB translocation and central nervous system infection. These results further emphasize the critical role of hemolysin-like proteins as key facilitators of neuroinvasive infections.

Beyond its role in direct tissue invasion, hemolysins have been implicated in modulating host immune responses. For instance, in *Staphylococcus aureus*, alpha-hemolysin activates the NLRP3 inflammasome in human and mouse monocytic cells, leading to the release of pro-inflammatory cytokines and contributing to the inflammatory response during infection (23). In the context of *C. sakazakii*, the deletion of the Hly III gene resulted in significantly reduced bacterial loads in brain tissue and a diminished inflammatory response, as evidenced by lower levels of pro-inflammatory cytokines and enzymes. This observation suggests that Hly III plays a crucial role in modulating the brain’s inflammatory response during infection.

A particularly interesting observation in this study was the enhanced desiccation resistance in the Δ*ESA_00432* mutant. Previous research has highlighted the complex relationship between desiccation resistance, biofilm formation, and surface hydrophilicity. Deletion of genes like *gsiD*, associated with glutathione transport, has been shown to decrease both desiccation resistance and biofilm formation simultaneously (24). Moreover, *C. sakazakii* G4023 demonstrated higher desiccation tolerance and more extensive biofilm formation than *C. sakazakii* ATCC 29544 (25), further supporting the role of biofilm in desiccation resistance. While some studies suggest that extracellular polysaccharides in biofilms, such as colanic acid, contribute to desiccation survival (26), our findings diverge in that Hly III, despite influencing desiccation tolerance, does not appear to play a role in biofilm formation.

Instead, our data suggest that desiccation resistance is more closely tied to surface hydrophilicity. Deletion of *ESA_00432* increased the hydrophilicity of *C. sakazakii*, which aligns with studies showing that hydrophilic surfaces contribute significantly to bacterial desiccation resistance (27, 28). This is evident from other examples, such as hydrophilins DtpA and DtpB in *Acinetobacter baumannii*, which enhance desiccation tolerance (27), or hydrophilins YJL144W and YMR175W in *Saccharomyces cerevisiae*, which aid both desiccation and freezing tolerance (29). Hydrophilic bacterial surfaces likely facilitate the retention of water during desiccation, providing a survival advantage under stress conditions.

These observations point to a broader trade-off between virulence and environmental resilience, a concept that has been observed in other bacterial species. In *Vibrio cholerae*, for example, virulence factors are downregulated under environmental stress to prioritize survival over pathogenicity (30). Similarly, in *Salmonella*, mutations that impair virulence factors often result in enhanced resistance to starvation (31). Consistent with these findings, this study also observed a decrease in Hly III gene transcription levels under desiccation stress. These findings highlight a common evolutionary strategy where survival mechanisms take precedence over virulence. Our study adds to this body of knowledge by demonstrating how Hly III, a virulence factor, plays a critical role in neuroinvasion. However, its absence leads to increased survival under desiccation stress, further emphasizing the context-dependent nature of bacterial fitness.

In conclusion, this study highlights the complex interplay between virulence and environmental resilience in *C. sakazakii*. Hly III is crucial for neuroinvasion, but its absence results in enhanced desiccation resistance, reflecting a broader evolutionary trade-off. These findings provide important insights into the mechanisms by which bacterial pathogens balance their survival strategies in both host and non-host environments. Furthermore, targeting Hly III could offer a novel therapeutic strategy for combating *C. sakazakii* infections, particularly those involving the central nervous system. However, given the observed trade-off between virulence and desiccation resistance, it is essential to assess the potential impact of Hly III inhibition on the bacterium’s ability to persist in dry environments, such as powdered infant formula. Future studies should explore the effects of pharmacological inhibition of Hly III on bacterial fitness in both host and environmental contexts, providing a clearer understanding of its feasibility as a therapeutic target.

## MATERIALS AND METHODS

### Bacterial strains, plasmids, and growth conditions

The bacterial strains, plasmids, and primers utilized in this study are listed in Table 1. Bacterial strains were stored in LB broth supplemented with 15% glycerol (vol/vol) at −80°C and revived on LB agar plates or in LB broth before experimental use. Antibiotics were incorporated as needed, with final concentrations of 100 µg/mL ampicillin to maintain plasmid selection or screen for recombinants. All primer sequences were designed based on the genome of *C. sakazakii* ATCC BAA-894 and synthesized by Sangon Biotech (Shanghai, China). In-frame deletion mutants were constructed using the pCVD442-based allelic exchange strategy (32). Briefly, approximately 800 bp upstream and downstream flanking sequences of the target gene were amplified by PCR, fused by overlap extension PCR, and cloned into the pCVD442 vector. Biparental conjugation was used to transfer the plasmid into *C. sakazakii*, and recombinants were selected on LB agar supplemented with 10% sucrose to counter-select against the vector. Positive mutants were confirmed by PCR and sequencing. For complementation experiments, the target gene, including its native promoter region, was cloned into the low-copy vector pACYC184 and transformed into the deletion mutant via electroporation (33). Electroporation conditions for *C. sakazakii* transformation included a field strength of 12.5 kV/cm, a resistance of 200 Ω, and a capacitance of 25 µF, with 1 µg of plasmid DNA used per transformation. Successful transformants were selected on LB agar containing appropriate antibiotics and verified by PCR and sequencing.

**TABLE 1:** Bacterial strains, plasmids, and primers used in this study

Strain, plasmid, or primer	Description	Reference
Strains		
WT	Wild-type *Cronobacter sakazakii* BAA-894	ATCC
Δ*ESA_00432*	Markerless deletion mutant Δ*ESA_00432*	This study
Δ*ESA_00,432-*C	*ESA_00432* complementation in Δ*ESA_00432*	This study
*E. coli* DH5α	Strain for construction	(34)
S17 lambda pir	Strain for construction harboring lambda pir	(34)
S17 lambda pir-Δ*ESA_00432*	S17 lambda pir harboring pCVD442-Δ*ESA_00432*	This study
Plasmids		
pACYC184	Low-copy plasmid	(35)
pACYC184- *ESA_00432*	*ESA_00432* complementation vector	This study
pCVD442	Suicide plasmid for markerless deletion	(34)
pCVD442-Δ*ESA_00432*	*ESA_00432* deletion plasmid	This study
Primers (5′-3′)		
pCVD442-fwd	GGCTGTCAGACCAAGTTTACTCATATATACTTTAGATTG	
pCVD442-rev	GCAGATACTCTTCCTTTTTCAATATTATTGAAGCATTTATCAGGGTTATTG	
Δ*ESA_00,432-*A	GAAAAAGGAAGAGTATATTCAGAATATCCTTCACAATG	
Δ*ESA_00432*-B	GACGAACCATGTGGCGACTCCGGT	
Δ*ESA_00,432-*C	ACCGGAGTCGCCACATGGTTCGTCTCTACATTCGCTGAGGATTG	
Δ*ESA_00432*-D	GATTAATTGTCAAGGCTATCTCAAAAGTCACCATCGCGCCGAAC	
*ESA_00432*-comp-fwd	GGTCTAGATGTATGTGATTGCTTTTAAAAAGG	
*ESA_00432*-comp-fwd	GGTCTAGAGAAGAAGCGTAAGCGTCTGATGGC	
Δ*ESA_00432-*E	GGAAAAACTAATTATTTCGCTGG	
Δ*ESA_00432* F	GGCAAAATGTGGAGCAACCTGC	

### Rat assay

One-week-old Sprague-Dawley rat neonates were used in this study and randomly assigned to groups infected with the WT, Δ*ESA_00432* mutant, or Δ*ESA_00432* complemented strain. Each rat neonate was intragastrically administered with 1 × 10^8^ CFU of the respective strain in 20 µL of sterile PBS (1). Survival was monitored over 7 days, with observations made twice daily. Twenty-four hours post-infection, rat neonates were euthanized, and tissues including blood, liver, spleen, and brain were collected aseptically. Blood samples were directly plated for CFU determination, while tissues were placed in thick-walled centrifuge tubes containing a 3 mm steel bead and washed three times with 0.5 mL cold PBS (4°C) to remove surface contamination. The tissues were homogenized using a high-speed homogenizer at 5,000 rpm for 40 seconds to generate uniform suspensions. Homogenized samples were serially diluted 10-fold in PBS, and 50 µL of each dilution was spread onto LB agar plates, followed by incubation at 37°C for 24 hours. CFUs were counted on plates containing 30–300 colonies, and bacterial loads were calculated by multiplying the CFU count by the dilution factor. For neuroinvasion assays, bacterial loads in brain tissues were determined as described above, while inflammatory markers, including TNF-α, IL-1β, IL-6, COX-2, and iNOS, were quantified using enzyme-linked immunosorbent assay kits (Jiancheng, China) according to the manufacturer’s instructions, with absorbance measured at 450 nm using a microplate reader (Bio-Rad, USA).

### Adhesion and invasion assay

To evaluate the impact of the *ESA_00432* gene on cellular adherence and invasion, bacterial strains were tested using human intestinal epithelial cells (Caco-2) and HBMECs (36). For adherence assays, bacteria were mixed with confluent cell monolayers at a multiplicity of infection of 1:100 and incubated at 37°C in a 5% CO₂ atmosphere for 45 minutes. Following incubation, the cells were washed three times with PBS to remove non-adherent bacteria. Adherent bacteria were released by lysing the cells with 1% Triton X-100 (Abcone, China). The lysates were serially diluted 10-fold in PBS, plated on LB agar, and incubated at 37°C for 24 hours to quantify CFUs. For invasion assays, the protocol was similar to that of the adherence assay but included a gentamicin protection step to eliminate extracellular bacteria. After the initial 45 minute incubation for bacterial adherence, cell monolayers were treated with gentamicin (100 µg/mL) for 1 hour to kill extracellular bacteria. The cells were then washed three times with PBS to remove residual gentamicin and lysed using 1% Triton X-100. Intracellular bacteria were quantified by plating serial dilutions of the lysates on LB agar, followed by incubation at 37°C for 24 hours.

### Desiccation resistance assays

Desiccation resistance was tested using a modified silica gel-based desiccation model (35). Bacterial cultures in LB broth were harvested during the logarithmic growth phase by centrifugation, and the supernatant was discarded. The bacterial pellets were then resuspended in either PBS or 10% (wt/vol) formula milk powder. Aliquots of 100 µL were dispensed into 96-well plates, which were sealed and placed in a sterilized desiccator with silica gel to maintain low humidity. After initial viable counts were determined by serial dilution and plating, the samples were desiccated. Following desiccation, the samples were rehydrated with PBS, serially diluted, and plated onto (Plate Count Agar) medium (Hopebiol, Qingdao, China). To determine survival after desiccation, 100 µL of PBS was added to each dry sample and incubated at room temperature for 5 minutes. Bacterial cells were then suspended by pipetting, serially 10-fold diluted in PBS, and inoculated onto PCA agar plates. After overnight incubation at 37°C, bacterial colonies were counted.

### Biofilm formation assay

The biofilm-forming ability of *C. sakazakii* wild type, Δ*ESA_00432* mutant, and complemented strains was assessed using crystal violet staining (1). Overnight bacterial cultures grown in LB broth at 37°C with shaking at 200 rpm were adjusted to an optical density corresponding to approximately 10⁷ CFU/mL. A 100 µL aliquot of each suspension was transferred into 96-well polystyrene plates, with each strain tested in triplicate. The plates were incubated under static conditions at 37°C for 48 hours to allow biofilm formation. After incubation, the supernatants were gently removed, and the wells were washed three times with 0.9% NaCl to remove planktonic bacteria. To fix the biofilm, 200 µL of 99% methanol was added to each well and left for 15 minutes before air-drying. Subsequently, 200 µL of 0.1% crystal violet solution was added and incubated for 30 minutes to stain the biofilm. The excess stain was rinsed off with 0.9% NaCl, and the bound crystal violet was solubilized with 200 µL of 95% ethanol. Absorbance at 570 nm was measured using a microplate reader (Bio-Rad, USA) to quantify biofilm formation.

### RT-PCR

Reverse transcription PCR (RT-PCR) was performed using the CFXConnect system (USA, Bio-Rad) according to standard methods. The primer pair targeting the Hly III gene used in the RT-PCR assay was forward, 5′-CAGGCCGTGGACAGCAATGCC-3′ and reverse, 5′-CTGCCGCCATAGAGGCTGTAAC-3′. The primers targeting 16S rDNA, used as an internal control, were forward, 5′-CCTGGCTTTGAGCAGGGCTGG-3′ and reverse, 5′-TTCGACGCAGCCCTGTGCCGAG-3′. 16S rDNA was used for normalization.

### Cell surface hydrophobicity

The hydrophilicity of WT, Δ*ESA_00432* mutant, and complemented strains was assessed using a modified xylene-water phase separation assay (37). Bacterial cultures were grown in LB broth at 37°C with shaking at 200 rpm until reaching the mid-exponential growth phase. The cells were harvested by centrifugation at 4,000 × *g* for 5 minutes at 4°C, washed twice with sterile PBS, and resuspended to an OD_600_ of 1.0 (denoted as H_₀_). Aliquots of 2 mL from each suspension were transferred to glass tubes, and 0.4 mL of xylene was added to each tube. The mixtures were vigorously vortexed for 2 minutes to allow phase interaction and then incubated at room temperature for 2 hours to permit complete phase separation. After incubation, the OD_600_ of the aqueous (water) phase was measured (denoted as H). The hydrophobicity was calculated using the formula: H = (H_0_ − H)/H_0_.

### Statistical analysis

Statistical analyses were conducted using GraphPad Prism 9 software to determine the significance of differences between experimental groups. All experiments were performed independently at least three times to ensure data reliability and reproducibility. Differences among multiple groups were assessed using one-way analysis of variance (ANOVA) followed by Tukey’s multiple comparison test. Survival curve analyses were performed using the log-rank (Mantel-Cox) test. Data are presented as mean ± standard deviation and statistical significance was set at *P* < 0.05.

## Data Availability

All data generated and analyzed in this study are provided within the article. No additional datasets were generated that require public deposition.
